# Supplementary material for: Are carbon emissions trading and green financial instruments synergistic? -Comprehensive quantitative research based on content analysis
Source: PLoS One. 2024 Mar 7;19(3):e0298601. doi: 10.1371/journal.pone.0298601 (PMC10919601; doi:10.1371/journal.pone.0298601)
Supplement: S1 Data — (ZIP) [file pone.0298601.s001.zip › Data/地级市Z_F工作报告.zip!/─■╧─/╣╠╘¡╩╨/└┤╫╘╣1⁄2╓┌║┼í╛┬φ┐╦╩2╛▌═°í┐_2016.docx]

各位代表：

　　我代表市人民政府向大会报告工作，请与《固原市国民经济和社会发展第十三个五年规划纲要(草案)》一并审议，并请市政协委员和列席人员提出意见。

　　2015年及“十二五”工作

　　过去的一年，市人民政府认真贯彻落实自治区党委、政府和市委的决策部署，积极适应经济发展新常态，从严从实抓扶贫、统筹协调促发展。一是紧盯项目稳增长。完成地区生产总值217亿元，增长8.5%;全社会固定资产投资345.8亿元，增长15%;地方公共财政一般预算收入和支出分别完成15.91亿元和184.59亿元，分别增长4.02%和11.58%。开工建设500万元以上项目497个，其中60个重点项目开工58个。宁夏中南部城乡饮水安全工程又让20万人喝上泾河水。彭青高速、西毛一级公路建成通车，东毛、固西等高速公路今年投运。造林31万亩。建成博物馆应急避难广场、固原游乐园等市民休闲项目，城市建成区扩大到44.1平方公里;实施了一批大县城扩容提质工程;建设5个中心镇和35个美丽村庄。全市城镇化率达到43.1%。固原入选“宽带中国”示范城市。争取国家和自治区各类补助资金174.07亿元，国家专项建设基金项目48个12.9亿元。六盘山国际旅游休闲度假区项目完成审核报批。引进实施招商引资项目181个，到位资金146.9亿元，增长10%。二是聚焦民生促和谐。15件民生实事全面完成。制定了“1+21+X”脱贫攻坚规划，完善“财政+金融+产业+扶贫”金融扶贫机制，创建了“一平台、一模式、一协会、一体系”金融模式;启动实施两个带头人、劳动力素质提升、金融扶贫等扶贫工程和“9+3”免费中等职业教育计划，贫困村销号126个，脱贫7.8万人。完成10万平方米薄弱学校校舍改造和19所幼儿园建设，原州区、彭阳县义务教育均衡发展工作通过自治区验收。市医院迁建工程主体完工;在全市推广“先诊疗后付费”服务模式。改造农村危房危窑4.63万户、城市棚户区8642户。新增城镇就业7613人;转移农村劳动力30.9万人，创收46.2亿元。完成城镇居民人均可支配收入21144.1元，增长7.5%;农村居民人均可支配收入7002.1元，增长9.5%。三是深化改革显活力。在全区五市率先公布市级权责清单，推行“先证后照”“一证一码”联审联办，办理时限缩短80%。创建工程招投标“六分离”机制。进一步理顺市区城市管理体制。与平凉等周边城市建立定期沟通联系机制。完善金融服务体系，石嘴山银行固原分行挂牌运行;与国开行、农发行、宁夏黄河农村商业银行签订战略性合作协议，达成300多亿元协议贷款。年末金融机构存贷款余额分别达到359.15亿元、253.31亿元，存贷比达到70.53%，提高4.92个百分点。

　　2015年受经济下行影响和主观努力不够，GDP、投资、财政收入虽然完成了2015年11月市人大常委会调整后的增速，但主要指标均未完成市三届人大三次会议确定的预期目标，其中城乡居民和财政收入增幅离年初目标差距较大，其他指标接近完成。尽管如此，地区生产总值、第二产业增加值、第三产业增加值、工业增加值、固定资产投资、农民人均可支配收入、社会消费品零售总额等七项指标增幅居全区五市第一，也不影响“十二五”规划目标的顺利完成。五年来，经济年均增长11.1%，投资年均增长25.2%，财政收入和支出年均分别增长24.78%和18.6%，城乡居民收入年均分别增长10.1%和15%，主要经济指标均比2010年翻一番。城镇登记失业率3.71%，人口自然增长率9.49‰，居民消费价格涨幅1.9%，万元GDP能耗1.1吨标煤，这四项指标均完成自治区下达的任务。总体看，“十二五”时期，全市重点工作有亮点，难点任务有突破，城乡面貌变化大，综合实力提升快。

　　一、扶贫开发温暖人心。累计争取扶贫资金45.1亿元，完成贫困村销号189个，贫困人口由50.1万减少到26.7万。一是饮水、道路、住房大改善。解决了100万人的安全饮水问题。累计新修等级公路、农村公路5215公里，实现行政村通沥青(水泥)路。改造危窑危房10.46万户。县内移民全面完成，县外移民完成91.4%，累计搬迁移民4.9万户20.9万人。二是产业、投入、信心大提升。实施“5·30”及其倍增产业扶贫计划。建立“合作社+基地+贫困户”产业扶贫模式，用财政扶贫担保基金1.8亿元撬动银行贷款16.8亿元。争取国家部委牵头扶贫和区内单位定点帮扶，落实帮扶资金1.66亿元;争取福建援助资金1.3亿元，带动福建社会各界投资31亿元。基础改善，收入增加，增强了贫困群众脱贫致富的信心。

　　二、生态保护深得人心。生态成为我市一张靓丽名片。一是地更绿。实施退耕还林、天然林保护、城乡环境大绿化等工程，累计造林192.4万亩，森林覆盖率达到22.2%，高于全区9.69个百分点。建成人民公园、原州古雁、西吉北山、隆德龟山、泾源卧龙山、彭阳茹河6个市民休闲森林公园，城镇建成区绿化覆盖率达到36.69%。二是水更清。淘汰马铃薯小型加工企业175家，建成城市污水处理厂4个、农村污水处理设施37个、农村垃圾处理场42个。农用残膜回收率达到85%。渝河、葫芦河水质稳定达标排放。治理小流域26条1500平方公里。三是天更蓝。推广清洁能源，实施集中供热工程，取缔燃煤锅炉、茶浴炉446台，开展粉尘污染治理，空气质量优良天数保持在88%以上，居全国地级市前列。我市被确定为全国生态文明示范工程试点市和生态保护与建设示范区。

　　三、产业发展稳定人心。累计安排财政资金2.1亿元，推进产业优化升级，产业结构由2010年的30.9∶14.8∶54.3调整为2015年的20.8∶27.2∶52。一是农业特色彰显。沿五大河流域形成草畜、马铃薯、冷凉蔬菜、中药材、苗木五大特色产业带。全市肉牛饲养量突破100万头，马铃薯种薯达到28万亩，冷凉蔬菜达到58.7万亩。承办了第六届中国宁夏园艺博览会暨六盘山冷凉蔬菜节、第十届中国牛业发展大会;六盘山农产品品牌荣获自治区著名商标;我市被授予中国“冷凉蔬菜之乡”“马铃薯种薯之乡”和“六盘山黄牛之乡”。耕地稳定在503.8万亩，粮食总产实现“十连增”。农业增加值年均增长5.4%。二是工业载体形成。建成固原经济技术开发区和圆德慈善产业园、吉德慈善产业园、隆德六盘山工业园、泾源轻工产业园、彭阳王洼产业园，进驻企业553家，带动就业2.1万人。金昱元100万吨真空制盐等项目投产，王洼至原州区运煤铁路、王洼煤矿千万吨级矿区改扩建等项目进展顺利。工业增加值年均增长19.1%。三是服务业健康发展。须弥山石窟、火石寨国家地质森林公园晋升为国家4A级景区，建成泾河源旅游小镇、隆德老巷子、彭阳茹河瀑布等新景区。旅游人数和收入年均增长20.28%和30.36%。荣膺“最美中国·特色魅力旅游目的地城市”“全国十佳生态休闲旅游城市”和“中国绿色生态旅游城市”称号。西兰银综合物流园一期、固原宾馆、西港航空酒店等建成运营，新华百货等大型商业入驻。服务业增加值年均增长9.7%。

　　四、民生改善凝聚人心。累计办理民生实事108件，民生投入占财政支出80%以上。一是教育文化利民。新建、改扩建校舍313万平方米，农村义务教育学生营养改善计划全面落实，建立从学前到高等教育全覆盖的资助体系，全市高考上线率居全区前列。推进文化固原建设，实现乡(镇)有文化站、村有文化室，广播电视户户通和农家书屋村级全覆盖。乡村文化大院成为特色品牌。成功举办了三届全国群众登山健身大会暨宁夏六盘山登山节。二是医疗卫生便民。实现村村有标准化卫生室，在全区率先建立市级统筹城乡居民医疗保险制度、试行人人享有基本医疗卫生服务和城乡居民大病医疗保险政策。三是社会保障惠民。建立被征地农民养老保险制度;加强城乡低保规范管理，全市13.5万户21.6万人纳入城乡低保;泾源“老饭桌”模式在全区推广。四是就业住房安民。累计发放创业小额担保贷款14.3亿元，新增城镇就业39400人;转移农村劳动力153万人(次)，创收167.6亿元。建成保障性住房28364套161万平方米。2012年成功创建自治区园林城市，2014年启动“五城联创”活动，2015年实现自治区卫生城市和双拥模范城市的创建目标。

　　探索创新社会治理模式，群众安全感连续五年居全区五市第一。民族团结、宗教和顺的局面进一步巩固。国防动员、双拥优抚工作深入开展。统计、档案、气象、地震、人防、外事侨务、地方志、工青妇、残疾人等事业取得新的进步。

　　五、改革开放鼓舞人心。完成政府机构改革，政风行风建设成效明显。一是改革开放有突破。推进行政审批制度改革，市级审批事项从208项精简为57项，行政事业性收费从369项精简为39项，年均减收1.1亿元。改革投融资机制，引进社会资本参与城市天然气、游乐设施等建设和经营。城市公交改制工作走在全区前列。基本完成农村土地确权登记工作，推行林权抵押贷款试点。加强与港澳台、沿海发达省区和周边地市的交流合作。招商引资到位资金累计达到515亿元，年均增长40%。二是政府效能有提升。扎实开展党的群众路线教育实践活动、“三严三实”专题教育和“思想大解放、树立新形象”等系列活动，持续整改“四风”问题，干部的精神状态、工作作风有了新转变，政府效能有了新提升。2013年、2014年获全区五市效能目标管理考核一等奖。建成全市电子政务办公系统，政府会议减少20%、文件减少53.8%。开展电视问政40期，公开政务信息22.5万条，办理政民互动信件11587件。区市人大代表167件议案、建议，政协委员878件提案全部办结。三是依法行政有实效。健全依法决策机制，出台了政务公开、公共资源交易管理、财政资金管理、行政效能问责、机关事业单位工作人员遴选办法等16项制度，行政行为进一步规范。“六五”普法工作顺利通过自治区验收。完善政府债务审批偿还机制，做好政府性债务防范化解工作，全市五年累计举债134亿元，偿债81亿元，负债率40%，债务率90%，均在风险防控标准之内;建立“农民工工资保证金”“应急周转金”和企业“拖欠黑名单”等制度，保障了农民工合法权益，维护了诚信政府形象。严格落实党风廉政建设责任制，加强对生态移民、保障性住房、扶贫及涉农资金、政府投资项目的审计监督和行政监察，民主评议政风行风活动群众满意度稳步上升。

　　各位代表，过去的五年，成绩令人鼓舞、变化催人奋进。我市生态建设、民族团结、产业扶贫在全国创造了经验，生态旅游、绿色农业、乡村文化大院在西部形成了优势，“三苦”作风、公共资源交易方式创新、“老饭桌”模式在全区打造了亮点，特别是扶贫开发取得的巨大变化，受到了习近平总书记的充分肯定。在此，我代表市人民政府，向各位代表和委员，向全市各条战线上的劳动者、各民主党派、工商联、人民团体、社会各界和中央、自治区驻固单位、人民解放军、武警官兵及公安干警，向所有关心支持固原建设和发展的朋友们，表示崇高的敬意和衷心的感谢!

　　五年的实践，也积累了珍贵的经验。一是坚持市委领导、人民监督是政府依法行政的根本保证;二是解放思想、大胆实践是政府推动发展的前提条件;三是项目建设、产业升级是政府扶贫开发的重要手段;四是改善民生、依法治理是政府维护稳定的坚实基础;五是发展经济、保护生态是政府为民尽责的中心任务。这些经验是全市人民探索出的宝贵财富，我们将倍加珍惜，发扬光大。

　　回顾“十二五”，发展短板依然突出。一是创新活力不足。鼓励创新的机制不健全，一些干部思想因循守旧，谋事眼界不宽，干事方法落后，创新精神欠佳。引进培养人才的机制不健全，高学历、高技术人才短缺，创新型、专家型人才稀缺。政府人员法治意识淡薄，依法行政能力亟待提高。二是发展基础不强。一方面，经济发展质量不高，产业层次低、链条短，产品进入高端市场少;居民增收来源单一，增速放缓;占经济总量半壁河山的服务业中新兴服务业仅占10%。另一方面，对外开放程度低，支撑开放的重大基础设施项目、经营性项目很少;互联互通的高等级公路少、民航航线少，快速铁路空白。三是脱贫机制不活。经济总量小，财政自给率低，金融服务体系不完善。贫困人口劳动技能低，自我脱贫的内生动力不足;市县整合扶贫资源、资金、项目的机制不健全，全社会参与扶贫的氛围不浓厚。对此，我们必须采取超常措施加以解决。

　　“十三五”目标任务

　　“十三五”时期是我市脱贫摘帽、同步小康的决胜期，更是我市补齐短板、拔掉穷根的机遇期。从全国看，国家实施“一带一路”倡议、“互联网+”行动计划和推进供给侧改革，有利于拓展我市开放合作空间，培育新的经济发展动力;中央向贫困宣战，出台一系列“滴灌式”脱贫政策，有利于加快我市补齐短板、改善发展条件。从全区看，深化宁夏建设，为我市对外开放搭建了大平台、构建了大通道;我市作为全区脱贫攻坚的主战场和宁南区域中心城市建设的主阵地，将会得到更多的政策、项目和资金倾斜支持，推动城乡协调发展。从我市看，业已形成的产业基础、生态资源、地域文化、“三苦”作风，是我市坚实的物质和精神财富，尤其是市委三届七次全会进一步明确了方向，凝聚了共识，必将激励全市人民积极投身全面脱贫、同步小康的伟大实践。我们必须坚定信心不动摇、咬定青山不放松，用好机遇，顺势而为。

　　按照市委三届七次全会总体要求，今后五年的发展思路：认真贯彻落实党的十八大和十八届三中、四中、五中全会、中央扶贫开发工作会议精神及自治区党委、政府和市委的决策部署，围绕“四个全面”战略布局，牢固树立创新、协调、绿色、开放、共享的发展理念，以脱贫攻坚为统领，夯实交通、水利、教育、生态、城镇化、信息化“六个基础”，实施劳动力素质提升、两个带头人、产业提质增效、民生改善、旧城改造、金融扶贫“六大工程”，深化改革开放，坚持项目带动，加强依法治理，维护社会稳定，全面从严治党，确保到2018年实现贫困人口脱贫、贫困县全部摘帽的目标，努力建设固原，奋力夺取全面建成小康社会的胜利。

　　发展目标：经济增长与自治区同步、民生发展与自治区同步;生态建设、社会建设在全区争先。到2020年，实现地区生产总值350亿元，年均增长8%;全社会固定资产投资600亿元，年均增长12%以上;地方公共财政一般预算收入25亿元，年均增长9%;城镇居民人均可支配收入35000元，年均增长10%;农村居民人均可支配收入12000元，年均增长11%;人口自然增长率控制在10‰以内。

　　实现上述目标任务，全市人民必须大力弘扬“不到长城非好汉”的六盘山精神，转变发展理念，创新工作方法，千方百计搭桥造船，同舟共济破浪前行。

　　一、精准脱贫，产业富民。脱贫是我市发展最根本的任务，既是压力和责任，更是机遇和希望。全面贯彻自治区脱贫攻坚誓师大会精神，统筹解决好扶持谁、谁来扶、怎么扶、如何退的问题，让贫困户早日过上好日子。

　　(一)全面落实“1+21+X”脱贫攻坚规划。实施精准脱贫“五个一批”计划，发展生产和转移就业脱贫12.7万人左右，易地搬迁脱贫4万人左右，生态补偿脱贫1万人左右，发展教育脱贫2万人左右，社会保障兜底脱贫7万人左右，坚决打赢脱贫攻坚战。一是落实好领导责任。脱贫是党政一把手工程，要逐级签订责任状，做到一个规划、一名责任领导、一个主抓部门、一套落实措施、一批脱贫项目，如期脱贫一批人。二是实施好扶贫工程。落实“六个精准”，夯实“六个基础”，抓实“六大工程”，创建精准扶贫、精准脱贫、产业致富的扶贫开发固原经验。三是发展好扶贫机制。发扬“三苦”作风，构建全社会扶贫大格局，深化闽宁协作、部门定点帮扶、社会力量参与扶贫开发工作。5年整合建立扶贫担保基金和风险金20亿元，撬动银行贷款200亿元以上，用好扶贫再贷款和支农再贷款政策，扶持发展特色产业。再造产业支撑、社会帮助、金融撬动、政策托底的扶贫新机制。

　　(二)高标准构建特色产业新模式。用市场是发展最稀缺资源的理念，选项目、强产业。一是围绕“3+X”模式发展现代农业。草畜产业，按照科技支撑、龙头带动、市场主导的要求，扶持培育一批养殖专业合作社、养殖大户和精深加工龙头企业，组建六盘山草畜产业集团和肉牛交易中心。到2020年，全市牧草基地稳定在300万亩，肉牛饲养量达到120万头，精深加工率达到60%以上。林业林下经济，坚守503.8万亩耕地红线，到2020年，林木种苗稳定在40万亩，林下经济达到125万亩，建成西北特色苗木基地、油用牡丹种植基地和特色林下经济示范基地。高标准建成60万亩高效节水农业示范区。把六盘山绿色农产品打造成中国驰名品牌。旅游产业，按照全域旅游的思路，突出生态休闲特色，打响“天高云淡六盘山”品牌。建成旅游环线、六盘山国际旅游休闲度假区、六盘山红军长征景区提升等项目，创建国家级红色旅游经典景区。到2020年，接待游客1000万人次，旅游收入突破80亿元以上。二是积极发展新型工业。按照生态、环保、节水的理念，整合市区现有工业园区，建立综合服务体系。适度发展能源、铝型材等新型产业，完成金昱元盐化工循环经济园、王洼煤矿千万吨级矿区改造、华电新能源及装备制造等项目;打造重要的特色农副产品加工贸易基地、轻工产品制造基地。新入驻企业300家。三是重点发展现代服务业。顺应生活消费快速增长、需求升级的大趋势，发展文化旅游、商贸物流、金融保险、健康养老、社区服务等业态，引导服务业向专业化和高端化迈进;加快西兰银物流园区建设。服务业占GDP保持在50%以上。

　　二、创新驱动，项目带动。创新是发展最关键的动力。实现思维方式由政府主导向市场决定转变，行为方式由要素拉动向创新驱动转变，发展路径由依赖资源向依靠科技转变，是我市“十三五”适应新常态的必然选择。

　　(一)培育发展新动力。健全促进创新的体制机制，激发市场活力和社会创造力。一是突出科技创新、科技引领。创新政府投入机制，支持企业加快技术改造，研发推广新技术、新产品、新模式;健全农业科技服务体系，推进固原国家农业科技园区建设，围绕全域和特色产业搭建技术研发平台;扶持科技人员领办创办实体和开展创业服务，让科技在全产业链、全领域支撑引领发展。二是激励大众创业、万众创新。实施“互联网+”创业创新行动计划，落实积极的创业就业政策，鼓励发展众创、众包、众扶、众筹空间。实施六大创业行动带动就业，年均新增城镇就业1万人左右，转移农村劳动力30万人(次)左右。三是加强人才引进、人才培养。人是发展最核心的要素。制定骨干人才补贴奖励政策，引进一批产业、金融、旅游、信息技术、城市管理等急需人才;实施本土技能型、实用型人才培养计划。

　　(二)补齐短板强基础。补短板是我市推进供给侧改革的主要任务。加强项目前期工作，提高项目编制质量，积极主动与国家部委、自治区厅局对接，促成一批重大项目进入规划，建设一批、投产一批重大基础设施、产业、民生项目;还要根据国家政策走向和我市需要，再谋划一批、储备一批，力争补齐短板。一是交通提标。开工建设银昆高速甘城至高寨塬段，打通向东连接庆阳、向南连接宝鸡、向西连接兰州等高速断头;改造提升农村公路2000公里。争取兰州经定西、固原、平凉至庆阳快速铁路和宝中铁路扩能改造、固原火车站综合枢纽中心项目早日开工建成。扩建六盘山机场，增加到东部大城市的航线，实现县县通高速，城际通高铁，现代综合运输体系初步建成。二是水利提效。落实最严格的水资源管理制度，完善促进节约用水的水价形成机制。以实施六盘山集中连片特困地区扶贫开发水资源利用工程为抓手，新建、改造中小型水库27座、中型以上淤地坝208座，年供水能力达到2亿立方米，形成北引黄河水、南引泾河水、利用库井水的“智能水网”。三是信息提速。建成“智慧固原”应用管理系统，实施城乡光纤入户工程，实现宽带网络全覆盖，全市家庭宽带普及率达到85%以上，让城乡居民共处信息时代，共享信息资源。

　　三、生态优先，协调发展。生态是发展最宝贵的优势。认真落实《宁夏空间发展战略规划》和《清水河城镇产业带总体规划》，优化生态、农业和城镇空间布局，让生态更良好、城市更宜居、乡村更美丽。

　　(一)保护好天高云淡、绿树成荫的美丽环境。把六盘山建成国家重点生态功能区和生态补偿试验区，把固原建成全国生态文明示范区。实施天然林保护、新一轮退耕还林还草、移民迁出区生态修复等工程，再造林100万亩，治理流域1600平方公里。森林覆盖率达到27%以上。加大禁牧封育、林木管护工作力度，巩固提升生态建设成果。严格环保执法，深入实施水、土壤、大气污染防治行动计划，加大饮用水源地保护力度;加强城镇污水处理设施建设和稳定运行，淀粉加工等工业废水达标排放;综合治理清水河、葫芦河、渝河、茹河水环境，持续改善水质。空气质量优良天数稳定在90%以上。

　　(二)建设好宜居宜业、功能完善的新型城镇。推进国家新型城镇化综合试点和“海绵城市”建设工作，建成“1411”城镇体系。到2020年，消除城市棚户区，争取政策解决自发移民(迁徙)有关问题，户籍人口城镇化率提高到45%(常住人口55%)。实施旧城改造工程，完成老城区1.8万户220万平方米的棚户区改造，综合改造老旧小区;建成西南新区，完善新区功能，提升区域中心城市的辐射带动能力。实施山水园林绿化工程，形成“四大主题公园”“四条生态山脉”“四大水库湿地”等市民休闲新空间。推行城市精细化管理，创建自治区文明城市、水生态文明城市和国家园林城市。加快县城产城融合发展，增强承载创业就业能力。把三营、兴隆、沙塘、泾河源、王洼等10个中心镇培育成商贸型、旅游型、工矿型特色镇，带动周边农村居民就地城镇化。沿旅游环线、五河流域布局建设或改造提升100个美丽村庄。

　　四、共建共享，改善民生。满怀真情做好民生工作，坚持保障水平只增不减，民生标准只升不降，用政府“紧日子”换百姓“好日子”。

　　(一)真诚服务百姓。增加公共服务供给，保障基本民生，提高公共服务共享水平。一是教育让百姓求学更公平。大力发展学前教育，新(改)建幼儿园110所，建立农村贫困家庭儿童学前教育补贴制度。改善义务教育薄弱学校条件，实现义务教育均衡发展。普及高中阶段教育。全面实施“9+3”免费中等职业教育计划，提升市民族职业技术学院和各县职业中学办学水平。深入推进教育改革，促进教育公平发展，做好关心下一代工作，让孩子快乐学习，健康成长。二是医疗让百姓看病更放心。深化公立医院综合改革，争取市人民医院创建三甲医院，完成县乡村三级医疗卫生服务网络标准化建设，创建群众满意的基层医疗卫生机构;健全城乡大病救助机制，让群众看得上病、看得起病、看得好病。密切与知名医院、著名专家多形式的务实合作，让医疗专家在家门口给群众看病不再是一种梦想。三是文化让百姓生活更丰富。实施文化惠民工程，建设市文化馆、图书馆、丝路文化展览馆和县(区)文化体育场(馆)，公共文化体育设施免费开放。培育发展文化产业，做实做靓文化大院品牌。加强非物质文化遗产开发和保护，丝绸之路(宁夏固原段)申遗成功。四是保障让百姓心里更踏实。实施全民参保计划，实现低保政策与扶贫政策相衔接，建立贫困户大病医疗救助制度。加强养老服务体系建设，完成固原市养老服务中心、农村敬老院等项目。

　　(二)厚植和谐根基。弘扬优良传统，融入法治理念，集聚新固原正能量。一是筑牢精神高地。以各类文明创建为统揽，接地气、增素质、提精神、强关爱，培育更多“固原好人”，创建更多文明家庭，涌现更多道德模范，关爱更多农村留守儿童和妇女、老人，激发更多善心善举，让热爱固原、建设固原成为全社会自觉行动。二是永续“平安固原”。建立社会治安信息化立体防控体系，完善社会矛盾排查预警和调处化解综合机制，有效预防和化解矛盾纠纷。开展“七五”普法，增强全民尊法学法守法用法意识。依法打击各类犯罪，维护社会秩序，群众的安全感和幸福指数进一步提升。三是提升应急能力。提高政府有效应对自然灾害、事故灾难等突发事件的处置能力。完善安全生产责任和管理制度，加大监管执法力度，坚决遏制重特大安全事故发生。强化食品药品监管，确保群众饮食用药安全。四是增进民族团结。加强民族团结宣传教育，深入开展民族团结进步创建活动，营造“六盘儿女一家亲”的浓厚氛围;依法管理宗教事务和活动，促进宗教领域和谐稳定，创建全国民族团结进步模范市。

　　五、开放引领，深化改革。坚持走出去开阔眼界、解放思想，请进来输入先进理念、先进经验。

　　(一)加快开放步伐。开放是拓展发展空间的必由之路。抢抓“一带一路”机遇，主动融入丝绸之路经济带战略节点城市建设，拓展经贸文化合作，建成中阿文化旅游合作产业园。积极承接中东部适宜产业转移。在工业园区、金融、旅游等领域合作上取得突破性进展。深化闽宁对口协作，筹办好闽宁协作20周年活动。加强与陕甘宁蒙毗邻地区的对接联系，联合争取国家重大政策和项目支持区域发展。用好海峡两岸(宁夏)经贸文化交流、中阿博览会等平台，着力引进一批科技含量高、投资强度高、项目产出高、产业辐射带动能力强的龙头企业。强化签约项目跟踪服务，五年引进资金1000亿元以上，年均增长15%左右。

　　(二)加快改革进程。“十三五”发展的任务重、矛盾多，关键要靠改革。重点在发展急需、民众企盼的主要领域和关键环节，推进落实一批改革任务。深化行政审批制度改革，推行权力清单和责任清单，推进集中审批和网上办理;创新政府管理方式，强化事中事后监管。深化农村综合改革。完成户籍制度改革，促进农业转移人口进城落户。深化投融资体制改革，财政资金重点向培植财源、建立贷款担保基金、支持民生事业、人才培养方面倾斜;加快县(区)融资平台建设，市县(区)政府通过争取中央专项基金、发行债券、采用PPP模式、银行政策性贷款等渠道，累计筹措项目建设资金200亿元，撬动金融机构贷款和社会资本800亿元。扩大金融机构覆盖面，推动金融服务体系向基层延伸。落实去杠杆政策，全市累计偿还、化解、置换政府债务100亿元。防范和化解金融风险，规范和监督民间借贷行为，打击非法集资活动，维护经济秩序。

　　各位代表，绘蓝图鼓舞斗志，奔小康信心满怀。只要我们不懈奋斗，固原的穷根就一定会拔掉，固原建设的步伐一定更稳健。

　　2016年重点工作

　　2016年各项工作的顺利开局，为“十三五”脱贫攻坚、同步小康按下了快进键。完成地区生产总值增长8%、全社会固定资产投资增长12%、地方公共财政一般预算收入和支出分别增长8%和10%以上、城镇和农村居民人均可支配收入分别增长9%和10%、社会消费品零售总额增长8%的目标以及自治区下达的4项约束性指标，需要我们艰苦奋斗，众志成城，合力攻坚以下六项重点工作。

　　一、抓实精准脱贫规划。安排1亿元扶贫开发担保资金，整合投入16.5亿元，完成150个贫困村销号，10万人脱贫。一是实施劳动力素质提升工程。整合各类培训资源，实施技能培训3万人，其中1.8万人取得国家职业资格证书。每个县(区)发展10个技能培训示范村、创建1个有规模、带动力强的创业孵化园。发放就业创业贷款4.2亿元以上，新增城镇就业9000人，城镇登记失业率控制在4%以内。转移农村劳动力30万人以上，创收53亿元。二是实施“两个带头人”工程。每个县(区)发展2个示范乡镇、15个示范村，每个村培育3—5名增收致富典型，发挥引领示范作用。三是实施民生改善工程。认真办理自治区民生计划和我市10项20件民生实事(见附件2)。全面完成“十二五”县外移民任务，启动“十三五”生态移民工作。加快固原八中建设，完成固原二中和30所幼儿园改扩建工程，改组并扩建五原中学。争取在贫困村实行学前3年教育“双补”试点。继续实施免费中等职业教育，改扩建市民族职业技术学院。完成530所标准化村卫生室温暖工程，市医院迁建工程上半年投入运行。加快丝绸之路申遗步伐，完成市文化馆、图书馆、丝路文化展览馆主体工程;新建4个乡镇文化站，扶持发展20家示范文化大院。建成固原市养老服务中心及信息平台，新建“老饭桌”151个。实行城乡居民大病保险扩面提标，全面落实被征地农民养老保险制度。推进低保政策与扶贫政策有效衔接。实施计划生育政策，人口自然增长率控制在10‰以内。四是实施金融扶贫工程。整合建立扶贫产业担保和风险补偿基金5.6亿元，撬动银行贷款30亿元。争取中央专项基金20亿元、国开行和农发行等棚改项目贷款40亿元，利用PPP模式融资15亿元。积极协调宁夏银行在县(区)设立分支机构，引进区内外银行发起设立村镇银行，消除金融服务农村“空白点”。

　　二、抓实产业提质增效。统筹安排1亿元产业引导扶持资金，建基地、引龙头、创品牌，加速产业提质增效。一是草畜产业突出抓点示范、节本增效，发展万头以上肉牛养殖示范乡镇5个、千头以上肉牛养殖示范村10个，开工建设万家灯火现代草畜产业及光伏扶贫等项目，肉牛饲养量达到108万头。二是林业林下经济坚持因地制宜、规模发展，建设油用牡丹基地6万亩、林业产业示范点20个，发展特色经果林8.8万亩、苗木1万亩、林下种植6万亩、林下养殖19.6万只。扶持发展高效绿色农业，建立高原冷凉蔬菜标准化示范基地25个，蔬菜种植面积达到60万亩;创建万亩标准化种薯示范基地10个，种薯繁育26.5万亩;中药材达到18万亩。农业增加值增长5%左右。三是旅游产业围绕整合资源、完善体制，推进智慧旅游建设，实施六盘山国际旅游休闲度假区、六盘山红军长征纪念馆改造提升，固原至张易、张易至隆德、西吉至火石寨、老龙潭至凉殿峡4条旅游环线和泾源县城至老龙潭20公里旅游精品线路等项目。力争全年接待游客突破400万人(次)，旅游社会收入30亿元。开工建设固原公益性农产品批发市场，扶持发展电子商务、邮政快递等新业态。服务业增加值增长8%以上。工业重点理顺市区工业园区管理体制，完善“一区五园”基础配套。实施金昱元盐化工产业链、华电风力发电、新中天特色农产品加工等项目。新增规上工业企业5户。工业增加值增长10%以上。

　　三、抓实重点项目建设。全年计划实施500万元以上项目460个，总投资1200亿元，当年投资450亿元，突出抓好9个重大工程包30个重点项目(见附件1)。继续开展“准备之冬、开工之春”活动，安排项目规划设计、环评等工作经费5000万元，加快推进土地报批、征地拆迁、招投标等前期工作，争取新建重点项目4月份全部开工。全年计划争取各级财政安排的项目资金100亿元，金融机构贷款和社会资本135亿元，招商引资到位资金165亿元，保障项目建设顺利推进。交通方面，建成东毛、固西高速和彭阳至高寨塬等8条公路，实现县县通高速。争取开工建设S70线彭阳至麻子沟圈、S25线泾源至双疙瘩梁高速断头路和轻工产业园至硝口一级公路。新(改)建农村公路500公里。积极做好宝中铁路扩能改造和兰州经定西、固原、平凉至庆阳快速铁路等项目前期工作。水利方面，中庄水库调试供水，改造新建沈家河、寺口子等中小型水库20座;启动固原市高效节水示范区建设，实施抗旱应急水源工程19处;开工建设清水河固原城区段治理工程，治理小流域15条312平方公里。生态方面，推进水源涵养区、生态修复区、退耕还林区等区域造林绿化33.4万亩。实施中庄水库流域环境综合治理等工程。争取立项建设六盘山生态功能区保护工程。完成城乡大环境绿化2万亩、道路及沿线绿化200公里，建成3000亩以上集中绿化点5个。做好污染防治工作，改善环境质量。空气质量优良天数达到90%以上。信息化方面，建设智慧固原，与自治区同步建成“八朵云”。推进“宽带中国”示范城市建设，完成624个贫困村农村宽带网建设，4G网络信号实现全覆盖。实施“互联网+农村扶贫电子商务”工程，提升5个县级电商配送中心，建成220个农村电商服务站。

　　四、抓实旧城改造任务。完成《老城区修建性详规》，实施棚户区改造、市政设施提升、老旧小区整治、古城风貌恢复、绿化亮化、综合管理六大任务。改造棚户区8600户110万平方米，改造提升老旧小区20个16万平方米，新改建道路18条20公里，配建排水、集污管网31条31公里。开工建设古城墙遗址公园、固原文化产业城和清水河国家湿地公园，完成九龙公园、会议中心周边等市区绿化100万平方米。实施西吉县中科广场、隆德县市政基础设施、泾源县商贸综合体、彭阳县茹河大街延伸段等工程，提升城市承载力。建设黄铎堡、将台、杨河、泾河源、古城5个中心镇和15个美丽村庄。改造农村危房危窑1.7万户。数字化城市管理信息系统投入运行，推行城市网格化管理模式。争创自治区文明城市和水生态文明城市，启动创建国家园林城市。

　　五、抓实发展环境优化。深化重点领域改革，扩大开放合作，创新社会治理，打造“两优”环境，建设服务政府。完成30项改革任务。推进精准扶贫机制创新、农村金融扶贫、农村土地股份制、供销社、农村户籍制度、小型水利工程管理体制、创新林权抵押贷款机制和农村基层党组织建设“八项改革试点”，为全区提供经验。深化行政审批和执法改革，组建市级行政审批局，探索建立综合执法局。完成公车改革，推进事业单位分类改革。出台人才培养引进意见，市财政安排3000万元，推动人才工作、基层组织和村干部队伍建设。改革市区、旅游、工业园区等管理体制。落实与平凉、庆阳等周边地区合作协议。推动安全生产和公共安全标准化信息化建设，开通“网上信访大厅”，推广基层矛盾纠纷排查调处“134”模式和诉前调解机制。加强道路交通、森林防火、食品药品等重点领域、重点行业的安全隐患排查和整治。深入开展民族团结“三化”“四重”“五联系”创建活动;创新宗教事务管理模式，维护现有宗教格局稳定。

　　六、抓实工作作风改进。践行“三严三实”要求，解放思想抓落实、突出重点抓落实、苦干实干抓落实、压实责任抓落实，建立市级重大项目、市领导重点工作、部门重要工作、县(区)主要任务四个责任清单，完善考核办法，强化督查问效，干出在全区有影响、有特色的亮点工作。坚持依法行政，主动接受市人大依法监督和政协民主监督，认真办理人大代表议案建议和政协委员提案，落实人大决议和政协民主协商计划;自觉接受社会和舆论监督。充分发挥工会、共青团、妇联等群团组织的作用。支持公检法司依法行使职权。支持国防和部队建设，促进军民融合发展。加大行政效能问责力度，着力解决乱作为、不作为、慢作为、怕担当等问题。推进诚信政府建设，年内偿还政府债务30亿元，其中市本级10亿元。严格落实党风廉政建设责任制，不折不扣执行“八项规定”，严控“三公”经费。下决心解决群众身边的不正之风和涉农资金管理中存在的公开不够、农民知道的少，监管不力、农民拿到的少，查处不严、农民信任的少等问题。进一步推进政务村务公开，自觉接受群众监督。强化政府投资项目、扶贫资金等重点领域的行政监察和审计监督，严肃查处违纪违法案件，营造风清气正的良好生态。

　　各位代表，用实干书写历史，用业绩回报人民。让我们紧密团结在以习近平同志为核心的党中央周围，在自治区党委、政府和市委的坚强领导下，凝心聚力，精准发力，创新苦干，为实现如期脱贫、与全区同步建成小康社会目标而努力奋斗!
